# How safety leadership influences employee safety participation and compliance through safety knowledge: the moderating role of psychological resilience

**DOI:** 10.3389/fpsyg.2025.1615084

**Published:** 2025-12-01

**Authors:** Juanxiu Piao, Juhee Hahn

**Affiliations:** 1 The Graduate School, Chung-Ang University, Seoul, Republic of Korea; 2 Department of Business Management, Chung-Ang University, Seoul, Republic of Korea

**Keywords:** safety leadership, safety knowledge, psychological resilience, safety compliance, safety participation, hierarchical linear modeling

## Abstract

**Introduction:**

This study developed a multilevel model to examine the mechanisms that link safety leadership to employee safety behaviors. We investigated (a) the mediating role of individual safety knowledge in the correlation between team-level safety leadership and employees’ safety compliance and participation, and (b) the moderating role of psychological resilience in this mediation process.

**Methods:**

We gathered three-wave survey data from 505 frontline employees and 88 supervisors within China’s electric power industry.

**Results:**

Hierarchical Linear Modeling (HLM) analysis confirmed that safety leadership positively influenced both safety compliance and participation. Employee safety knowledge significantly mediated this relationship. Furthermore, psychological resilience moderated this process: the positive effect of safety knowledge on both safety compliance and participation is stronger among employees with high psychological resilience.

**Discussion:**

These findings indicate that organizations should invest in safety leadership training to enhance employee knowledge and foster psychological resilience as a crucial personal resource, enabling employees to more effectively convert safety knowledge into safe behavior.

## 1 Introduction

Workplace safety has consistently been a significant concern in both developed and developing economies (Cioni and Savioli, 2016). The electric power industry is among the most hazardous sectors owing to its complex technologies, high voltages, and continuous production processes (Albert and Hallowell, 2013; Basahel, 2021). Despite advancements in safety systems, fatal incidents continue to pose a significant challenge. For instance, in 2020, China’s National Energy Administration reported 35 electric power-related personal injury accidents, resulting in 44 fatalities nationwide (National Energy Administration, 2021). These incidents highlight that human and organizational factors, rather than solely technical failures, are the principal contributors to workplace injuries and fatalities (Zohar and Polachek, 2014).

Recent research emphasizes that leadership is crucial in influencing workplace safety outcomes (Cheung et al., 2021; Vatankhah, 2021; Omidi et al., 2023). Safety leadership—defined as leaders’ ability to communicate, model, and enforce safety values—has been recognized as a key predictor of both compliance and participatory safety behaviors. However, empirical evidence elucidating how safety leadership translates into employees’ safety behaviors remains limited, particularly in high-risk and collectivist cultural contexts such as China (Mosly and Makki, 2020). Previous research has largely examined transformational or transactional leadership without fully clarifying the cognitive mechanisms underlying this process (Clarke, 2006; Mullen and Kelloway, 2009).

Recent studies have begun to specify these pathways. For example, safety leadership influences workers’ safety outcomes via safety climate, psychological contracts of safety, and risk perception (Omidi et al., 2023), underscoring the necessity to examine cognitive mechanisms such as safety knowledge. Additionally, longitudinal evidence indicates that safety climate is a stronger predictor of compliance than of participation (Syed-Yahya et al., 2025), reinforcing the necessity to model these two behaviors separately.

According to Social Learning Theory (Bandura, 1986), employees observe and imitate leaders’ safe behavior, internalize safety standards, and implement them in their daily activities. Thus, safety leadership fosters safety knowledge via role modeling and communication, thereby enhancing safety compliance and participation (Abdullah et al., 2020; Subramaniam et al., 2023). Furthermore, psychological resilience—employees’ ability to recover from stress and adapt to adversity—constitutes a key personal resource that determines how effectively individuals apply their safety knowledge (Lu et al., 2023). According to Conservation of Resources (COR) Theory (Hobfoll, 2011; Bakker and Demerouti, 2017), resilience enables employees to conserve and mobilize cognitive and emotional resources under strain. Empirical research substantiates this concept, demonstrating that resilience mitigates safety risks and enhances engagement in hazardous occupations (Ni et al., 2023).

To address these theoretical and empirical gaps, this study develops a multilevel model that integrates leadership and personal resource perspectives. Specifically, it examines (a) the mediating role of safety knowledge in the relationship between safety leadership and employees’ safety behaviors and (b) the moderating role of psychological resilience within this mediation framework. Survey data were gathered from 88 supervisors and 505 frontline employees across 88 workgroups within China’s State Grid Corporation. To analyze the data, this study applied hierarchical linear modeling (HLM).

This study contributes to the existing literature in three distinct ways. First, it enriches safety leadership research by identifying safety knowledge as a cognitive mechanism that links leadership to safety outcomes. Second, it extends COR theory by demonstrating how resilience amplifies the effect of knowledge on behavior in high-risk, resource-demanding contexts. Third, it offers practical insights for enhancing safety management in large state-owned enterprises, emphasizing the dual significance of leadership training and employee resilience development in fostering a proactive safety culture.

## 2 Literature review and hypotheses development

### 2.1 Safety leadership

Safety leadership refers to the implementation of leadership styles in safety management within organizations and delineates how leaders influence employees to attain elevated safety performance levels (Wu, 2005). Wu (2008) identified three dimensions: safety controlling, safety coaching, and safety caring. Safety controlling involves establishing regulations and monitoring compliance; safety coaching emphasizes role modeling, decision participation, and motivation; and safety caring reflects leaders’ trust and concern for employees’ well-being (Lu and Yang, 2010; Wu, 2008). According to holistic leadership theory, safety leadership comprises both transactional and transformational components (Oswald et al., 2022). Transactional safety leadership prioritizes compliance through monitoring and contingent reward, whereas transformational safety leadership motivates followers through vision, idealized influence, and safety-oriented values (Clarke, 2013). Empirical research indicates that leadership behaviors, such as intellectual stimulation, inspirational motivation, and recognition, correlate with reduced accident rates (Yule et al., 2007).

Recent evidence further indicates that safety leadership influences outcomes via proximal social-cognitive mechanisms (such as safety climate, psychological contracts, and risk perception), reinforcing the necessity to articulate “how” leadership translates into behavior (Omidi et al., 2023).

### 2.2 Safety behavior

Safety behavior is widely considered a primary determinant of workplace accidents (Yeow et al., 2020). It refers to employees’ adherence to safety procedures and their voluntary participation in safety-related activities (Hsu et al., 2010; Kapp, 2012). Griffin and Neal (2000) conceptualized two dimensions: safety compliance—obligatory actions to uphold workplace safety—and safety participation—voluntary behaviors that enhance a positive safety climate (Neal et al., 2000). Longitudinal research indicates that safety climate more robustly predicts compliance than participation, underscoring the value of modeling these behaviors separately (Syed-Yahya et al., 2025).

Because safety behaviors reflect psychological and motivational factors more than tangible outcomes, they serve as a sensitive indicator of safety performance (Xia et al., 2023).

### 2.3 Safety leadership and safety behavior

Leaders impact safety directly by demonstrating safety-oriented actions and indirectly by cultivating the organizational safety climate (Rahlin et al., 2022). Prior research confirms that robust safety leadership lowers accident rates and enhances employees’ compliance and participation (Lu and Yang, 2010; Levovnik et al., 2019; Xue et al., 2020). Leaders’ commitment to safety—through clear communication, frequent worksite visits, and active involvement—fosters trust and psychological safety among employees (Basahel, 2021). According to Social Learning Theory (Bandura, 1986), employees observe and emulate leaders’ safe behavior; according to Social Exchange Theory (Blau, 1964), supportive leader–member relationships encourage reciprocation via safety motivation and participation (Tucker et al., 2008; DeJoy et al., 2004; Zhang et al., 2020).

*H1*: Safety leadership positively affects employees’ safety compliance and safety participation.

### 2.4 Mediating role of safety knowledge

Safety knowledge refers to employees’ comprehension of safety procedures and hazard-control methods (Pyo, 2005). It enables individuals to identify risks, adhere to protocols, and engage in preventive measures (Zhang and Fang, 2013; Chua and Goh, 2004). Effective knowledge management enhances accident prevention and reduces injury frequency (Choudhry et al., 2007; Yu et al., 2021). Leadership is central in building this knowledge via training, feedback, and modeling (Abad et al., 2013; Zerguine et al., 2018; Fang et al., 2020).

Consistent with Social Learning Theory, recent studies in construction settings indicate that leadership support enhances safety learning and safety citizenship, offering a concrete knowledge-based pathway from leaders to employee safety behavior (Kadher et al., 2024). Simultaneously, safety leadership has demonstrated an impact on workers’ safety outcomes through climate/contract/risk-perception mechanisms, highlighting cognitive routes (Omidi et al., 2023).

*H2*: Safety knowledge significantly mediates the relationship between safety leadership and safety compliance.

*H3*: Safety knowledge significantly mediates the relationship between safety leadership and safety participation.

### 2.5 Psychological resilience

Resilience is the capacity to sustain or regain psychological well-being under adversity (Fletcher and Sarkar, 2013). In positive psychology, it is a developable personal resource that facilitates adaptive responses to stress and uncertainty (Luthans, 2002; Rutter, 2006; Kuntz et al., 2017; Sisto et al., 2019). Resilience enhances employees’ ability to sustain safe performance under pressure and fatigue, correlating with improved safety-related outcomes (Christian et al., 2009; Lu et al., 2023).

From the perspective of Conservation of Resources (COR) Theory (Hobfoll, 2011; Bakker and Demerouti, 2017), resilience enables individuals to mobilize and protect cognitive/affective resources, thereby increasing the likelihood of effectively employing safety knowledge in high-risk situations. Consistent with this logic, recent empirical research associates resilience with diminished unsafe behavior (via burnout mitigation) and enhanced safety engagement; cross-level evidence further indicates that psychosocial safety climate fosters safety behavior by mediating psychological resilience under safety-related stress (Zhao and Li, 2024).

Within our multilevel framework, team-level safety leadership functions as a contextual resource that shapes a shared safety environment and builds employees’ safety knowledge. Psychological resilience, in turn, determines how effectively individuals can convert this knowledge into concrete safety behaviors when facing operational pressures, time constraints, or competing performance demands. Accordingly, we conceptualize resilience as a boundary condition of the second stage of the indirect path from safety leadership to safety behavior, such that the cross-level influence of leadership on behavior via knowledge is stronger among highly resilient employees.

*H4*: Psychological resilience moderates the relationship between safety knowledge and safety compliance.

*H5*: Psychological resilience moderates the relationship between safety knowledge and safety participation.

### 2.6 Conceptual model

**Figure 1:**
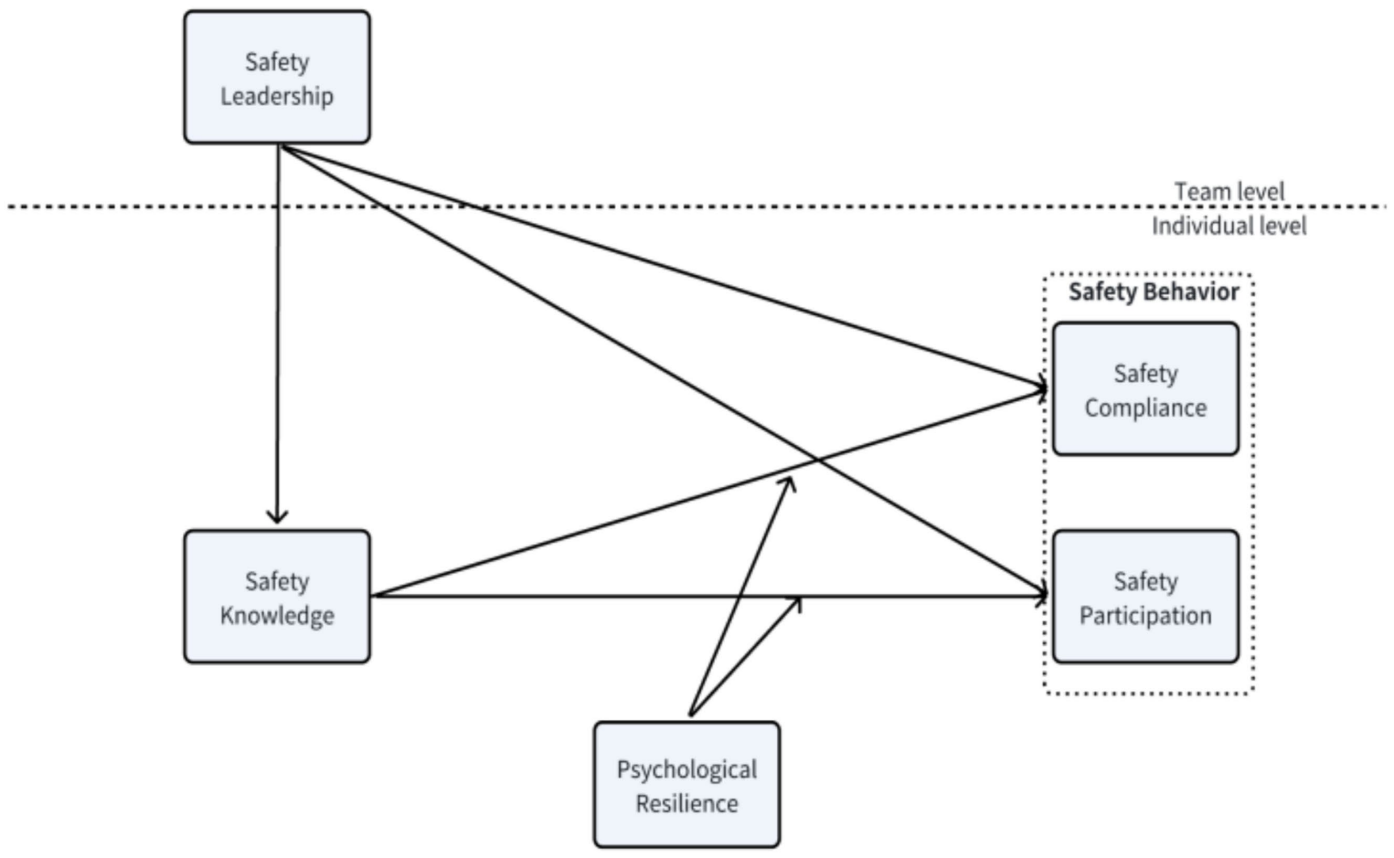
Research model.

The model (Figure 1) integrates leadership and personal resource perspectives. This study combines Social Learning Theory (Bandura, 1986) and Conservation of Resources Theory (Hobfoll, 2011), providing a coherent explanation of how leadership influences employee safety behavior through cognitive (knowledge) and personal (resilience) mechanisms. This results in a moderated-mediation account that links leadership, cognition, and personal adaptation in high-risk organizational settings.

More specifically, team-level safety leadership is expected to enhance individual safety knowledge, which in turn promotes safety compliance and participation at the employee level. Psychological resilience is proposed to moderate this second-stage link, such that the indirect (cross-level) effect of safety leadership on safety behavior via safety knowledge is stronger for employees with higher resilience. Thus, our model specifies a cross-level moderated mediation process that jointly incorporates leadership, cognitive resources, and personal resources.

## 3 Materials and methods

### 3.1 Sample and procedure

A purposive sampling strategy was employed to select five large state-owned power companies in China. Participants were assured of anonymity and confidentiality. These firms were selected because they are representative of China’s power industry regarding size, operational risk, and mature safety management systems, thereby offering a suitable context to test our hypotheses. Data were gathered from frontline employees and their immediate supervisors from three companies in Shandong and two in Beijing and Tianjin.

The Human Resources departments facilitated the sampling process by granting access to operational workgroups (teams). The inclusion criteria for employee participants were: (1) full-time frontline operational employees (i.e., excluding administrative staff) and (2) a minimum tenure of 6 months under their current direct supervisor, to ensure accurate assessment of their leader’s safety leadership.

To reduce common method bias and establish temporal separation among variables, data were collected in a three-wave design between March 11 and May 23, 2023. During Time 1 (March 11–21), employees rated their immediate supervisors’ safety leadership. At Time 2 (April 3–10), the same employees completed measures of safety knowledge and psychological resilience. At Time 3 (April 24–May 23), supervisors evaluated employees’ safety compliance and participation. The two- to three-week intervals between waves were chosen to reduce priming and recall effects while maintaining feasibility and minimizing participant attrition (Podsakoff et al., 2003). All measures were linked using anonymous codes to ensure confidentiality.

The selection of two- to three-week intervals follows methodological recommendations for reducing common method variance in longitudinal field studies (Podsakoff et al., 2003). Intervals of this length allow the effects of respondents’ initial ratings (e.g., leadership perceptions) to dissipate, thereby reducing priming, consistency motifs, and short-term affective carryover. At the same time, 2–3 weeks is short enough to ensure that relatively stable constructs (e.g., safety leadership, resilience) do not change substantially over time, and to minimize attrition and disruption to data linkage. Thus, the chosen intervals strike an optimal balance between reducing common method bias and maintaining construct stability.

The final sample comprised 505 employees nested within 88 teams (average team size ≈ 7). This sample size exceeds the minimum recommendations for sufficient statistical power in multilevel modeling (Maas and Hox, 2005). Descriptive statistics (SPSS 26.0) indicated that 69.3% of leaders were male, 59.1% were aged 31–50, and 54.5% held a bachelor’s degree or higher. Of the employees, 73.3% were male, 46.3% were aged 31–50, and 45.9% possessed over 7 years of experience—characteristics typical of China’s state-owned heavy industry workforce.

### 3.2 Measures

All measurement instruments were originally developed in English and subsequently translated into Chinese through a rigorous committee-based back-translation procedure (Brislin and Freimanis, 2001) to ensure both linguistic and conceptual equivalence. Each construct was assessed using a five-point Likert scale, with responses ranging from 1 (“strongly disagree”) to 5 (“strongly agree”). The complete questionnaire items are included in Supplementary Appendix A.

Safety leadership was measured at Time 1 using Wu’s (2008) 19-item scale, which captures safety coaching, caring, and controlling. Cronbach’s *α* = 0.933.

Safety behaviors were assessed by supervisors at Time 3 using the 12-item measure by Vinodkumar and Bhasi (2010), consisting of safety compliance (7 items; α = 0.917) and safety participation (5 items; α = 0.872).

Safety knowledge was measured at Time 2 with a six-item scale from Wang et al. (2016) (α = 0.898). Psychological resilience was measured at Time 2 using the seven-item scale validated by Ni et al. (2023) in Chinese industrial settings (α = 0.919).

### 3.3 Analytical strategy

Descriptive statistics were conducted using SPSS 26.0, while confirmatory factor analysis (CFA) was performed using AMOS 23.0. Hierarchical Linear Modeling (HLM 7.0) was employed to test all hypotheses, given the nested data structure of employees within teams.

To justify the application of HLM, we computed ICC (1) values for the Level 1 outcomes. The ICC (1) for safety compliance was 0.17 and for safety participation 0.21, indicating that 17 and 21% of the total variance in safety behaviors was attributed to the team level, respectively. These values exceed the 0.05 threshold recommended by Klein and Kozlowski (2000), thereby confirming sufficient within-group similarity and between-group variance to justify multilevel modeling.

We adhered to the multilevel moderated-mediation procedures established by Preacher et al. (2010). All Level 1 predictors (safety knowledge, psychological resilience, and their interaction) were group-mean centered, whereas the Level 2 predictor (safety leadership) was grand-mean centered. This centering approach facilitates the clear interpretation of cross-level effects and the distinction between within-group and between-group variance. The reliability and validity of all constructs were verified through CFA before conducting hypothesis testing (see Table 1).

**Table 1:** Scales’ reliability and validity.

Variable	Items	Alpha	Factor loading	CR	AVE
Safety leadership	19	0.933	0.713–0.833	0.961	0.569
Safety knowledge	6	0.898	0.744–0.819	0.898	0.595
Safety compliance	7	0.917	0.739–0.831	0.919	0.620
Safety participation	5	0.872	0.729–0.950	0.873	0.579
Psychological resilience	7	0.919	0.773–0.870	0.921	0.624

## 4 Data analysis and results

### 4.1 Analytical approach and confirmatory factor analysis

Before hypothesis testing, we performed multiple statistical analyses to ensure the reliability and validity of the measures. Table 1 showed that all Cronbach’s alpha coefficients exceeded the recommended threshold of 0.70 (George and Mallery, 2003), indicating robust internal consistency. All factor loadings exceeded 0.50, indicating acceptable item reliability (Hair, 1998). Confirmatory factor analysis (CFA) was performed utilizing AMOS 23.0 to evaluate the measurement model’s convergent and discriminant validity.

At the team level, model fit indices were satisfactory (χ^2^ = 952.443, df = 610, χ^2^/df = 1.536, IFI = 0.975, TLI = 0.968, CFI = 0.970, RMSEA = 0.033).

At the individual level, the indices indicated good fit (χ^2^ = 444.917, df = 269, χ^2^/df = 1.654, IFI = 0.977, TLI = 0.974, CFI = 0.977, RMSEA = 0.036).

All average variance extracted (AVE) values ranged from 0.569 to 0.624 and exceeded 0.50, whereas composite reliabilities (CR) for all constructs exceeded 0.70 (Safety leadership = 0.961, Safety knowledge = 0.898, Safety compliance = 0.919, Safety participation = 0.873, Psychological resilience = 0.921) (Nunnally, 1978). These results substantiated both convergent and discriminant validity.

To confirm the statistical appropriateness of aggregating individual-level safety leadership ratings to the team level, we calculated the within-group agreement index (Rwg) and intraclass correlation coefficients (ICC(1), ICC(2)) (Klein and Kozlowski, 2000). The Rwg values for safety leadership and safety climate were 0.767 and 0.731, respectively, both exceeding 0.70. ICC(1) values exceeded 0.05, and ICC(2) values exceeded 0.50, with *F*-tests significant at *p* < 0.001, thereby affirming adequate within-group consistency and between-group variance.

Common method bias was assessed through Harman’s single-factor test; the first factor accounted for 26.3% of total variance, which was below the 40% threshold (Podsakoff et al., 2003). Multicollinearity diagnostics indicated all variance inflation factors (VIFs) < 5, thereby confirming the lack of collinearity issues.

### 4.2 Descriptive statistics

Table 2 showed the means, standard deviations, and bivariate correlations for all study variables. As anticipated, safety knowledge was positively correlated with psychological resilience (*r* = 0.206, *p* < 0.01), psychological resilience was positively associated with safety compliance (*r* = 0.174, *p* < 0.01), and both variables were significantly related to safety participation (*r* = 0.255, *p* < 0.01). These correlations preliminarily supported the hypothesized relationships.

**Table 2:** Means (M), standard deviations (SD), and correlations among study variables.

**(a) Individual-level variables**	**1**	**2**	**3**	**4**	**5**	**6**	**7**	**8**
1. Age								
2. Gender	0.092*							
3. Education	0.090*	0.242**						
4. Work experience	0.123**	0.076	0.357**					
5. Safety knowledge	−0.113*	−0.074	0.0196	0.183	(0.771)			
6. Psychological resilience	−0.021	0.031	0.002	0.261	0.206**	(0.790)		
7. Safety compliance	−0.051	0.0269	0.080	0.183	0.319**	0.174**	(0.787)	
8. Safety participation	−0.018	−0.007	0.043	0.437	0.334**	0.255**	0.614**	(0.761)

**(b) Team-level variables**	**1**	**2**	**3**	**4**	**5**	**6**
1. Team leader’s gender						
2. Team leader’s age	0.062					
3. Team leader education	−0.034	−0.141**				
4. Team leader work experience	−0.076	0.337**	−0.092*			
5. Team size	−0.092*	−0.103*	0.048	0.261**		
6. Safety leadership	−0.100*	0.121*	−0.125**	0.107*	−0.028	(0.754)

*N* = 505 for individual-level data, and *N* = 88 for team-level data. *p < 0.05, **p < 0.01. The square roots of AVE are presented along the diagonal.

### 4.3 Hypothesis testing

Given the nested data structure (employees nested within teams), Hierarchical Linear Modeling (HLM 7.0) was employed to test the hypotheses and account for between-team variance. All Level 1 variables (safety knowledge, psychological resilience, and their interaction) were group-mean centered, while the Level 2 predictor (safety leadership) was grand-mean centered (Preacher et al., 2010).

Hypothesis 1 posited that safety leadership positively influences employee safety behavior. As shown in Models 4 and 5 of Table 3, safety leadership significantly correlated with both safety compliance (*β* = 0.552, *p <* 0.001) and safety participation (*β* = 0.450, *p <* 0.001), thereby supported H1.

**Table 3:** Hypothesis testing.

Variables	SK	SC	SP	SC	SP	SC	SP	SC	SP
	Model 1	Model 2	Model 3	Model 4	Model 5	Model 6	Model 7	Model 8	Model 9
Level 1									
Intercept	3.524***	3.571***	3.631***	3.575***	3.635***	3.572***	3.632***	3.577***	3.636***
Gender	−0.196	0.037	0.112	0.025	0.076	0.065	0.131	−0.005	0.044
Age	−0.097*	0.032	0.007	0.009	−0.024	0.028	0.002	0.010	−0.022
Education level	0.068*	0.104*	0.086	0.116**	0.1006	0.102*	0.082	0.130**	0.118
Work experience	−0.102*	0.042	0.021	0.017	−0.010	0.038	0.016	0.012	−0.016
Safety knowledge		0.239***	0.295***			0.202***	0.255***		
Safety knowledge * Psychological resilience								0.186***	0.139*
Level 2									
Leader’s gender	−0.174	−0.009	0.105	0.012	0.098	0.047	0.143	−0.02	0.068
Leader’s age	0.161	0.014	−0.011	0.009	0.006	−0.023	−0.035	0.051	0.041
Leader education	0.036	−0.040	0.014	0.005	0.051	−0.003	0.042	−0.054	0.005
Leader work experience	0.183	0.118	0.102	0.131	0.134	0.094	0.087	0.150	0.151
Team size	−0.067	−0.152	−0.126	−0.168	−0.144	−0.155	−0.129	−0.162	−0.638
Safety leadership	0.047***			0.552***	0.450***	0.457***	0.329***		
*R*	0.385	0.456	0.536	0.469	0.560	0.455	0.536	0.449	0.545
Tau	0.282	0.377	0.235	0.315	0.215	0.301	0.197	0.460	0.324
Chi-square	415.446***	465.038***	282.987***	389.781***	255.744***	385.311***	247.198***	556.987***	356.804***
Deviance	1119.511	1212.710	1253.738	1211.625	1266.159	1196.970	1243.437	1220.138	1279.449

*p ≤ 0.05, **p ≤ 0.01, ***p ≤ 0.001. SK=Safety knowledge, SC = Safety compliance, SP=Safety participation.

Hypotheses 2 and 3 posited that safety knowledge mediates the relationships between safety leadership and the two dimensions of safety behavior. Model 6 indicated a significant indirect effect of safety leadership on safety compliance via safety knowledge (*β* = 0.202, *p* < 0.001). Model 7 also exhibited a similar mediation for safety participation (*β* = 0.255, *p* < 0.001). These findings supported H2 and H3.

**Figure 2:**
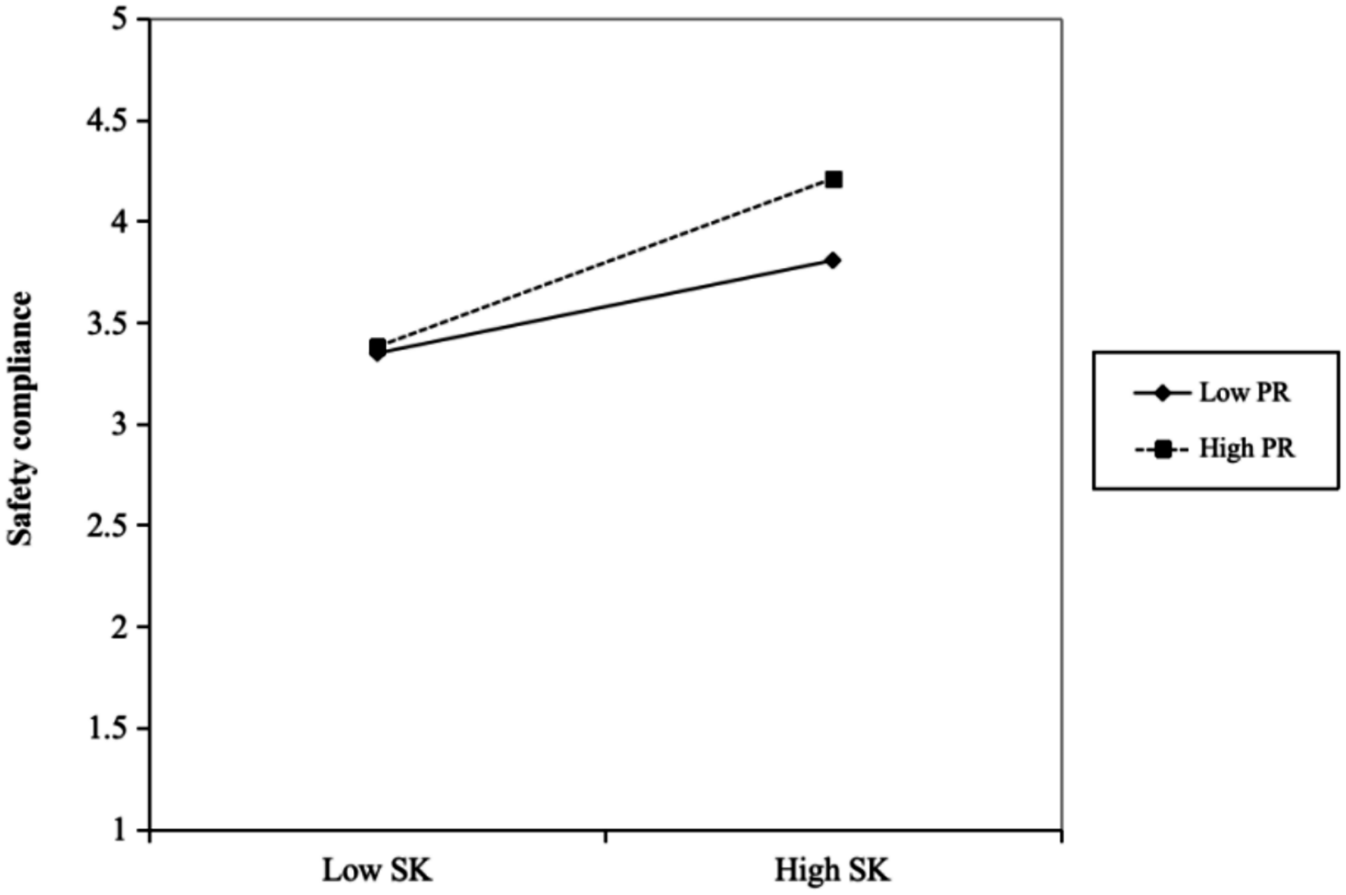
Moderating effect of psychological resilience on safety knowledge and safety compliance.

Consistent with H4, Figure 2 illustrated that psychological resilience significantly strengthens the positive relationship between safety knowledge and safety compliance. Specifically, the simple slope for employees with high resilience (+1 SD) was substantially steeper than that for those with low resilience (−1 SD). This pattern indicated that employees who are more psychologically resilient are better able to translate their safety knowledge into consistent rule-following behavior. In other words, resilience enhanced employees’ capacity to apply their safety-related cognitive resources under demanding or stressful work conditions, thereby amplifying the behavioral impact of safety knowledge.

**Figure 3:**
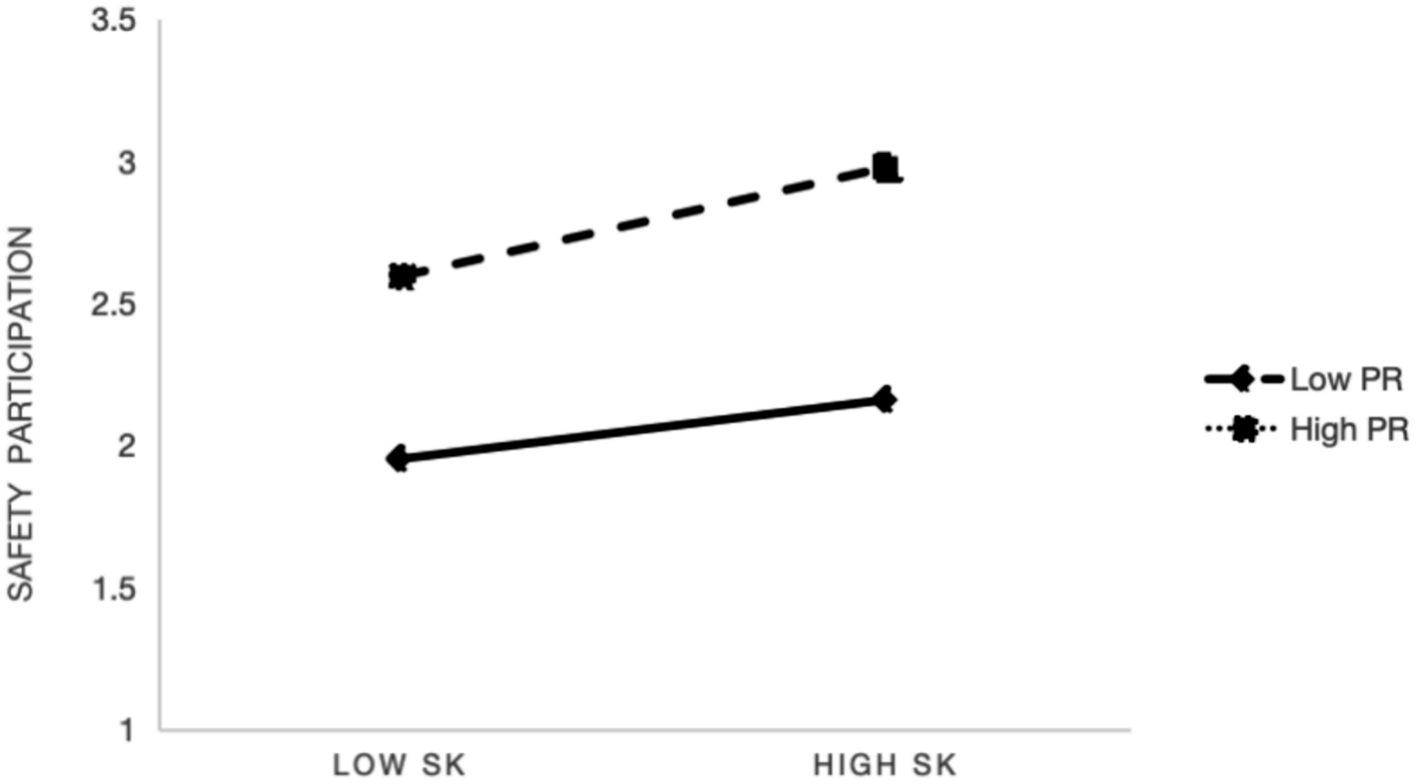
Moderating effect of psychological resilience on safety knowledge and safety participation.

Similarly, consistent with H5, Figure 3 showed that psychological resilience also moderates the relationship between safety knowledge and safety participation. The slope for employees with higher resilience was stronger compared to those lower in resilience, suggesting that resilient employees were more likely to convert their safety knowledge into proactive, discretionary behaviors such as making suggestions, assisting coworkers, or voluntarily engaging in safety activities. This supported the theorized role of resilience as a personal resource that facilitates the enactment of proactive safety behaviors beyond mandated compliance.

### 4.4 Moderated mediation

To formally test the proposed cross-level moderated mediation, we computed the index of moderated mediation (Preacher et al., 2007). For safety compliance, the IMM was significant (IMM = 0.0087), indicating that psychological resilience strengthened the indirect effect of safety leadership on safety compliance through safety knowledge. For safety participation, the IMM was also positive and significant (IMM = 0.0065), supporting the hypothesized moderated mediation.

We further examined the conditional indirect effects at low (−1 SD), medium, and high (+1 SD) levels of psychological resilience. Using a Monte Carlo simulation procedure with 20,000 repetitions (Selig and Preacher, 2008), all conditional indirect effects were statistically significant. For safety compliance, the indirect effects were 0.0369 (low resilience), 0.0406 (medium), and 0.0441 (high). For safety participation, the effects were 0.0325 (low), 0.0352 (medium), and 0.0379 (high). These results indicated that employees with higher psychological resilience more strongly translate their safety knowledge into both compliant and participatory safety behaviors, consistent with H4 and H5.

## 5 Discussion of findings

This study examined the multilevel mechanisms linking team-level safety leadership to individual-level safety behaviors in China’s electric power industry. The results confirmed that safety leadership significantly enhances employees’ safety compliance and participation, mediated by safety knowledge, while psychological resilience moderates these effects. Specifically, employees with higher resilience were more effective at translating their safety knowledge into compliant and participatory safety behaviors.

In interpreting the magnitude of the effects, several findings demonstrate meaningful and practically important relationships. First, the cross-level effect of safety leadership on safety compliance (*β* = 0.552) and participation (*β* = 0.450) represents moderate-to-large effect sizes within the context of multilevel safety research. These coefficients indicate that employees working in teams with stronger safety leadership practices exhibit substantially higher levels of both compliance and proactive participation. In practical terms, even incremental improvements in leaders’ safety communication, coaching, and monitoring may lead to sizeable improvements in frontline employee safety behaviors.

Second, the direct effect of safety knowledge on safety compliance (*β* = 0.202) and participation (*β* = 0.255) reflects small-to-moderate but meaningful behavioral effects. Given that safety behaviors are multiply determined and often constrained by situational demands, these coefficients demonstrate that improving employees’ safety knowledge has a reliable and practically relevant impact on behavior. This underscores the value of investing in safety training, structured knowledge-sharing, and operational briefings.

Third, the interaction terms for the moderating role of psychological resilience (*β* = 0.186 for compliance; *β* = 0.139 for participation) represent moderate cross-level interaction effects. These findings suggest that resilience functions as a leverage point: when employees possess higher psychological resilience, the behavioral benefits of safety knowledge become significantly stronger. Practically, this indicates that organizations may increase the effectiveness of safety knowledge by developing resilience-enhancing interventions—such as stress management, peer support mechanisms, or resilience workshops—especially in high-risk environments requiring rapid adaptation to hazards.

Taken together, the magnitude of the coefficients demonstrates that both leadership and individual resources meaningfully influence safety outcomes, and that investing in these domains may yield observable and impactful improvements in safety performance.

In summary, the results consistently demonstrate that safety leadership exerts significant cross-level effects on both safety compliance and safety participation, and that these effects operate partly through employees’ safety knowledge. The moderating role of psychological resilience further indicates that the translation of safety knowledge into behavior is stronger for more resilient employees. These statistical patterns collectively suggest a cognitively and motivationally integrated mechanism through which leadership influences safety performance. Building on these empirical findings, the following Discussion section elaborates on the theoretical and practical implications derived from these results.

### 5.1 Theoretical implications

First, this study extends Social Learning Theory (Bandura, 1986) by demonstrating how team-level leadership behaviors elicit reciprocal safety-oriented responses from employees. Leaders who actively communicate, model, and reward safe practices demonstrate organizational commitment to safety, prompting employees to reciprocate with increased safety compliance and participation. This finding reinforces prior evidence that safety leadership is a proximal determinant of safety behavior (Clarke, 2013; Wu et al., 2009; Cheung et al., 2021). Moreover, it contributes to leadership literature by specifying how such exchanges occur in high-risk, state-owned contexts—facilitated by enhanced safety knowledge as a cognitive mechanism.

Second, this study broadens existing leadership models by identifying safety knowledge as a mediator. Previous studies predominantly linked leadership to safety outcomes or performance (Kelloway et al., 2006; Lu and Yang, 2010), while few examined the knowledge-based pathway. Our findings indicate that when leaders prioritize safety coaching, monitoring, and caring, employees acquire more accurate operational knowledge, subsequently fostering safer behavior. This builds upon emerging research that emphasizes cognitive mechanisms in leadership–safety relationships (Vinodkumar and Bhasi, 2010; Abdullah et al., 2020; Fang et al., 2020) and provides an evidence-based justification for integrating safety knowledge management into leadership development frameworks.

Third, our study enhances the comprehension of psychological resilience within the realm of occupational safety research. Resilience—a developable psychological resource (Luthans, 2002; Fletcher and Sarkar, 2013)—moderated the relationship between safety knowledge and safety behaviors, particularly compliance. This asymmetry indicates that resilience exerts a more pronounced effect on rule-following behaviors compared to discretionary (participative) behaviors. This aligns with recent findings indicating that resilience promotes adaptive regulation during stress, thereby enhancing employees’ capacity to sustain safe performance despite external pressures (Lu et al., 2023; Kuntz et al., 2017). Taken together, these findings provide an incremental refinement of existing safety leadership and resilience models, by framing resilience as both a buffer and a facilitator in the knowledge–behavior conversion process in high-risk industry contexts.

### 5.2 Practical implications

From a managerial standpoint, the findings offer several actionable insights for enhancing safety management in high-risk sectors:

First, power companies should develop leadership training programs that prioritize communication, coaching, and trust-building to cultivate safety-oriented reciprocity. Managers who consistently model safe behavior, recognize employee contributions, and offer emotional support cultivate both safety knowledge and compliance.

Second, because safety knowledge mediates leadership effects, companies should integrate structured safety knowledge-sharing platforms and cross-departmental learning sessions. Regular workshops and scenario-based drills can enhance employees’ cognitive comprehension of hazards and emergency responses.

Third, because resilience enhances the translation of knowledge into behavior, organizations should incorporate psychological resource developments—such as stress management training and peer-support programs—into safety education. Moreover, managers can establish open, psychologically safe environments where employees feel secure discussing safety concerns and mistakes without fear of retribution (Zhang et al., 2020).

Fourth, although these implications are derived from China’s electric-power sector, they should be interpreted cautiously in broader contexts. State-owned enterprises in China function within hierarchical authority frameworks and collectivist principles, potentially amplifying the influence of leadership behaviors and moderating employees’ responsiveness to psychological interventions. In these environments, leaders’ role modeling and trust-based communication are not only managerial practices but also culturally anticipated behaviors that influence compliance. Future studies should examine whether similar multilevel mechanisms apply in private or international firms, where autonomy and safety climates significantly differ.

Finally, leaders should ensure two-way safety communication and implement reward systems that acknowledge not only compliance but also proactive participation, thereby sustaining long-term engagement in safety practices.

### 5.3 Limitations and future research directions

Despite its contributions, this study has several limitations.

First, the data were gathered from five large state-owned power enterprises in China, potentially limiting the generalizability of findings to private or international contexts. Future research should replicate the model across industries characterized by various ownership structures and regulatory regimes to examine boundary conditions.

Second, although purposive sampling was appropriate for accessing high-risk operational teams, the involvement of Human Resources departments in facilitating participant recruitment may introduce selection bias, as supervisors or HR staff might preferentially encourage certain employees or teams to participate. This may limit the generalizability of the findings. Future research should consider employing random sampling or independent recruitment channels to enhance representativeness and reduce potential sampling bias.

Third, although the three-wave, time-lagged survey design, which mitigates common method bias, is advantageous, it remains non-experimental and thus cannot conclusively determine causality. Consequently, future studies should employ longitudinal or experimental designs to more effectively capture temporal dynamics between leadership, knowledge, and resilience.

Fourth, only leaders evaluated employees’ safety behaviors, potentially introducing rater bias. Future research should employ multi-source assessments (e.g., peer or self-ratings) or objective safety metrics (e.g., near-miss data) to triangulate behavioral outcomes.

Finally, although this study concentrated on psychological resilience as a personal resource, other individual-level moderators—such as safety motivation, perceived organizational support, or safety climate—may also influence the interplay between knowledge and leadership. Future research should examine these additional variables to develop a more comprehensive multilevel model of safety performance.

## 6 Conclusion

This research presents an integrated, multilevel framework demonstrating that the impact of safety leadership is indirect; it is mediated through the employee’s cognitive resources (safety knowledge) and dependent upon their personal resources (psychological resilience).

By anchoring these mechanisms in Social Learning Theory and Conservation of Resources (COR) Theory, our findings shift the practical focus from mere compliance-monitoring to a more holistic approach: actively enhancing employees’ cognitive and psychological capacities. This resource-building perspective offers a robust framework for high-risk organizations to cultivate a more proactive and sustainable safety culture.

## Data Availability

The raw data supporting the conclusions of this article will be made available by the authors, without undue reservation.
