# Supplementary material for: How safety leadership influences employee safety participation and compliance through safety knowledge: the moderating role of psychological resilience
Source: Front Psychol. 2025 Dec 1;16:1615084. doi: 10.3389/fpsyg.2025.1615084 (PMC12702887; doi:10.3389/fpsyg.2025.1615084)
Supplement: Supplementary file 1 [file Supplementary_file_1.docx]

Supplementary Material

Appendix A

Employee Questionnaire: Safety leadership-Safety coaching

1.My managers handle safety business honestly.

2.My managers set an example by obeying safety regulations.
3.My managers help employees to recognize the importance of safety.

4.My managers explain the concept of safety clearly.

5.My managers involve personnel in safety decision-making.
6.My managers draw a picture to describe a safety vision.

Safety leadership-Safety caring

1.My managers create a harmonious group climate.
2.My managers allocate safety resources fairly.

3.My managers accept employees' advice to improve safety.

4.My managers be confident of employees' safety performance.

5.My managers make an effort to meet employees' need for safety.

6.My managers recognize employees' safety achievements.

Safety leadership -Safety controlling

1.My managers order employees to accomplish safety goals firmly.

2.My managers assess and reward staff safety performance effectively.

3.My managers support to establish regulations of safety management.

4.My managers request employees to obey regulations of safety management.

5.My managers request employees to improve safety defects continuously.

6.My managers audit employees' safety performance regularly.

7.My managers do not order employees to accomplish safety goals firmly.

Employee Questionnaire: Safety knowledge

1.I know how to perform my job in a safe manner.

2.I know how to use safety equipment and standard work procedures.

3.I know how to maintain or improve workplace health and safety.

4.I know how to reduce the risk of accidents and incidents in the workplace.

5.I know what are the hazards associated with my jobs and the necessary precautions to be taken while doing my job.

6.I don’t know what to do and whom to report if a potential hazard is noticed in my workplace.

Employee Questionnaire: Psychological resilience (PR)

1.I will refuse to work when appropriate preventive and protective measures are not provided.

2.I will refuse to work when it is not clear how to execute the work task.

3.I can aware the negative consequences resulting from noncompliance with health and safety rules.

4.I will remain vigilant to project hazards even when they are recognized and controlled with preventive measures.

5.I can judge and identify the potential hazards regarding work tasks.

6.I often pay attention to coworkers' activities and behaviors in work.

7.I will refuse to work when hazards and safety risks related to work task are not clear.

Leader Questionnaire: Safety Behavior-Safety compliance

1.This employee uses all necessary safety equipment to do his/her jobs.

2.This employee carries out his/her work in a safe manner.

3.This employee follows correct safety rules and procedures while carrying out his/her jobs.

4.This employee ensures the highest levels of safety when they carry out their jobs.

5.Occasionally due to lack of time, this employee deviates from corrects and safe work procedures.

6.Occasionally due to over familiarity with the job, my employees deviate from correct and safe work procedures.

7.It is not always practical to follow all safety rules and procedures while doing a job.

Leader Questionnaire: Safety Behavior-Safety participation

1.My employees help their co-workers when they are working under risky or hazardous conditions.

2.My employees always point out to the management if any safety related matters are noticed in our company.

3.My employees put extra effort to improve the safety of the workplace.

4.My employees voluntarily carryout tasks or activities that help to improve workplace safety.

5.My employees encourage their co-workers to work safely.
